# Avian top predator and the landscape of fear: responses of mammalian mesopredators to risk imposed by the golden eagle

**DOI:** 10.1002/ece3.1370

**Published:** 2015-01-05

**Authors:** Mari S Lyly, Alexandre Villers, Elina Koivisto, Pekka Helle, Tuomo Ollila, Erkki Korpimäki

**Affiliations:** 1 Section of Ecology, Department of Biology, University of TurkuFI-20014, Turku, Finland; 2 Centre d'Etudes Biologiques de Chizé UMR 7372, CNRS & Université de La Rochelle79360, Beauvoir sur Niort, France; 3 Finnish Game and Fisheries Research Institute, Oulu Game and Fisheries Research, University of OuluPOB 413, FI-90014, Oulu, Finland; 4 Finnish Forest and Park Services, Natural Heritage ServicesPOB 8016, FI-96101, Rovaniemi, Finland

**Keywords:** Intraguild predation, mesopredator suppression, pine marten, raptor, red fox, trophic interactions

## Abstract

Top predators may induce extensive cascading effects on lower trophic levels, for example, through intraguild predation (IGP). The impacts of both mammalian and avian top predators on species of the same class have been extensively studied, but the effects of the latter upon mammalian mesopredators are not yet as well known. We examined the impact of the predation risk imposed by a large avian predator, the golden eagle (*Aquila chrysaetos*, L.), on its potential mammalian mesopredator prey, the red fox (*Vulpes vulpes*, L.), and the pine marten (*Martes martes*, L.). The study combined 23 years of countrywide data from nesting records of eagles and wildlife track counts of mesopredators in Finland, northern Europe. The predation risk of the golden eagle was modeled as a function of territory density, density of fledglings produced, and distance to nearest active eagle territory, with the expectation that a high predation risk would reduce the abundances of smaller sized pine martens in particular. Red foxes appeared not to suffer from eagle predation, being in fact most numerous close to eagle nests and in areas with more eagle territories. This is likely due to similar prey preferences of the two predators and the larger size of foxes enabling them to escape eagle predation risk. Somewhat contrary to our prediction, the abundance of pine martens increased from low to intermediate territory density and at close proximity to eagle nests, possibly because of similar habitat preferences of martens and eagles. We found a slightly decreasing trend of marten abundance at high territory density, which could indicate that the response in marten populations is dependent on eagle density. However, more research is needed to better establish whether mesopredators are intimidated or predated by golden eagles, and whether such effects could in turn cascade to lower trophic levels, benefitting herbivorous species.

## Introduction

The traditional view of trophic dynamics has emphasized the importance of bottom-up effects in ecosystems, but recent studies have provided increasing evidence to show that top-down processes are also extensive and influential in shaping communities (e.g., Hebblewhite et al. 2005; Borrvall and Ebenman 2006; Terborgh and Estes 2010). A typical case of such process is top predators preying upon and limiting the numbers of herbivores. Indeed, top predators are often key species in their ecosystems and may induce extensive cascading effects on the lower trophic levels (Estes et al. 2011; Ripple et al. 2014).

Another pathway for top-down effects is intraguild predation (IGP), where predatory species compete over shared prey but also prey on each other (Polis et al. 1989; Lourenço et al. 2014). Among vertebrates, IGP is typically inflicted by larger species on their smaller competitors, the killer species being usually more than three times larger than the victim species (Palomares and Caro 1999; Sergio and Hiraldo 2008). In addition to direct killing and consumption, IGP often induces avoidance and risk-sensitive habitat selection in the prey, reducing their breeding success and survival (Mitchell and Banks 2005; Sergio and Hiraldo 2008; Mukherjee et al. 2009). Top predators may thus act as an important mortality factor for smaller predators (Ritchie and Johnson 2009; Pasanen-Mortensen et al. 2013) and generate a “landscape of fear” where mesopredators experience differing levels of predation risk (Laundré et al. 2001; Swanson et al. 2014). However, if top predator populations decline, they can cease to limit populations of mesopredators, as is suggested by the mesopredator release hypothesis (Soulé et al. 1988; Crooks and Soulé 1999; Prugh et al. 2009). For example, in Australia, the absence of a top predator, the dingo (*Canis dingo*, Meyer), has resulted in a mesopredator release, which in turn has caused devastating cascading effects on the small marsupial fauna (Johnson et al. 2007).

Most of the previous studies on vertebrate IGP have focused on within-class predator guilds (*mammal–mammal IGP*: Palomares et al. 1995; Courchamp et al. 1999; Helldin et al. 2006; Letnic et al. 2011; *bird–bird IGP*: Hakkarainen and Korpimäki 1996; Fielding et al. 2003; Sergio and Hiraldo 2008; Lourenço et al. 2011). For example, studies from North America show that the wolf (*Canis lupus*, L.) has a negative impact on the coyote (*Canis latrans* Say) not just via predation but also through intimidation and food competition (Berger and Gese 2007; Miller et al. 2012). Moreover, there is a clear interaction extending to the red fox (*Vulpes vulpes*, L.): when wolves are present, foxes are more numerous than coyotes, whereas when wolves are absent, coyotes dominate in numbers (Newsome and Ripple 2014). Nonetheless, observations from the prey remains of large raptors show that mammalian mesopredators succumb to predation by birds, too (Sulkava et al. 1997, 2008; Watson 2010). This suggests that large birds of prey could also affect the abundance and distribution of mammalian mesopredators. Yet, such predatory interactions have been reported only in a few articles (Korpimäki and Norrdahl 1989; Roemer et al. 2002; Moehrenschlager et al. 2007; Salo et al. 2008).

One of the largest raptor species in the Northern Hemisphere is the golden eagle (*Aquila chrysaetos*, L.). Many of its populations crashed during the early 20th century, largely due to persecution (Whitfield et al. 2004; Ollila and Koskimies 2006; Watson 2010). Currently golden eagle populations are mainly stable or increasing, but their recovery has been slowed down by habitat fragmentation and increased human disturbance (Watson 2010). The golden eagle is a top predator, which can prey over a wide range of species, from small birds, and rodents even to ungulates as large as deer. The main prey items come from the groups of hares (Leporidae), grouse (Tetraonidae and Phasianidae), and squirrels (Sciuridae) (Watson 2010). What differentiates golden eagles from many other raptors is the relatively high percentage of mammalian predators in their diet (Valkama et al. 2005; Lourenço et al. 2011), typically ranging from 2% to as much as 10–20% (Watson 2010). Therefore, the paucity or absence of the golden eagle may have contributed to the increase of mesopredators in many ecosystems during the past decades (Korpimäki and Nordström 2004).

The aim of this article was to expand the understanding of intraguild relationships between avian top predators and mammalian mesopredator prey. We do so by examining whether the Finnish golden eagle population impacts abundances of two carnivorous mesopredators, the red fox, and the pine marten (*Martes martes*, L.). Both species are common in Finland (Wikman 2010) and known to be preyed upon by eagles (ca. 1% of fresh prey remains at nest sites each, Sulkava et al. 1999). We combine two countrywide data sets gathered from Finland over several decades: the nesting inventory data of golden eagles and the wildlife snow-track census data, which as annual monitoring scheme of multiple species is unique in its extent and longevity. We use estimated eagle territory and fledgling densities together with distance to the nearest eagle nest as proxies of the predation risk, the impact of which we then examine on mesopredator abundances. However, the mechanism for such impact can be either direct predation, dispersal due to intimidation, or both, and the two phenomena cannot be set apart with observational data. Instead, we study the overall impact of eagles on mesopredator abundances. We predict that the abundance of the pine marten, similar in size with the main prey items of eagle, will be lower in areas where there are more active eagle territories and fledglings produced, and also in the vicinity of inhabited eagle nests. In contrast, we expect the larger red fox, which is at the upper end of the weight scale of eagle prey, not to respond to our proxies of predation pressure.

## Materials and Methods

### Golden eagle data

**Figure 1:**
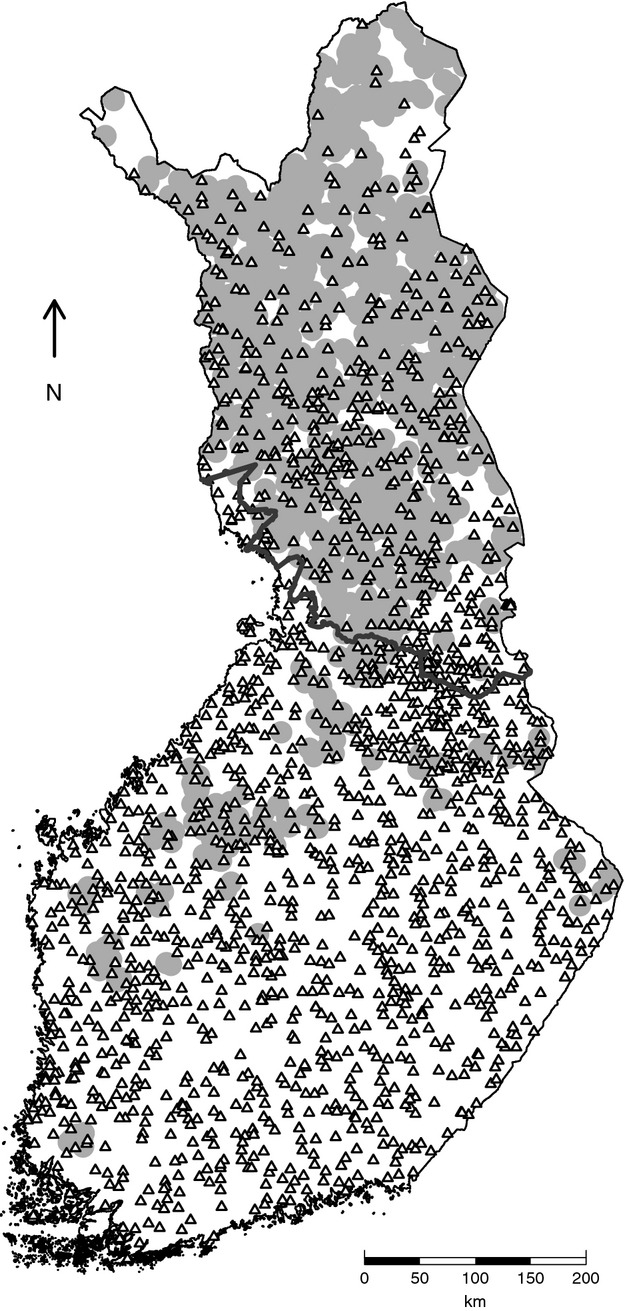
Map of Finland showing the locations of wildlife triangles (triangle symbols) and golden eagle nesting sites (grey area) included in the study. The nesting sites are presented with a randomized ≤10 km offset and a 10 km buffer zone. The southern border of reindeer husbandry area is presented with a dark grey line.

**Figure 2:**
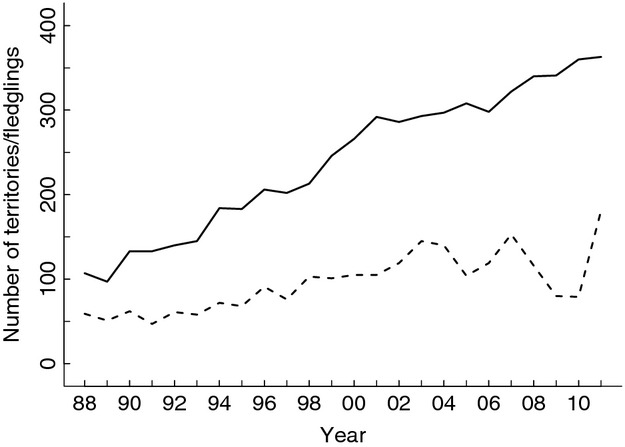
Population trends of the golden eagle during 1988–2011 in Finland. The number of active territories (solid line) and fledglings produced (dashed line) are shown annually.

In Finland, the golden eagle is dispersed over an area larger than 150,000 km² (Fig.1), mainly within the reindeer (*Rangifer tarandus*, L.) husbandry area in the northern parts of the country (Ollila and Ilmonen 2009). During the past decades, the number of breeding eagle pairs has been increasing (Ollila and Koskimies 2006; Fig.2), reaching 348 inhabited territories in 2014 (Ollila 2014). The nesting data of the golden eagle, provided by the Finnish Forest and Park Service, include information about nest occupancy and breeding success of eagles in Finnish nesting sites. The data are derived from an ongoing large-scale monitoring of all known territories and active searching for new ones, carried out annually by some 40 voluntary bird ringers.

In this study, we included all the known territories that had records of eagle presence during 1988–2011 (*n* = 477, 6569 individual records). Nests were annually categorized into two classes according to their breeding success: (1) unsuccessfully breeding pairs (present but not breeding, with possible records of a failed breeding attempt, such as broken egg shells or remains of chicks); and (2) successfully breeding pairs (present with ≥1 fledglings produced). To analyze the impact of golden eagles upon mesopredator species, we then used the eagle nest locations to form two types of annual eagle density maps. In the territory density (TD) maps, all active golden eagle territories were included (classes 1–2), whereas fledgling density (FD) maps were formed using only the nests with fledglings produced (class 2), multiplied with the number of fledged chicks (1 or 2). The obtained countrywide density maps reflect the predation pressure – both direct and indirect – by the golden eagle.

The eagle density maps were produced by computing a smoothed intensity function (raster size 1 km) from the nest locations, using the “density.ppp” function from the R package “spatstat” (Baddeley and Turner 2005). The density.ppp computes a kernel estimate (Diggle 1985) of the intensity function of a point process, which generated the pattern of nests (*u*). It computes the convolution of the isotropic Gaussian kernel with point masses at each data point in *u*: 

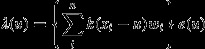
1

In the intensity function *λ*(*u*), the points in the neighborhood of a single point *u* are given by *x*_*i*_ and their weights by *w*_*i*_, equaling to 1 in our case. The edge correction factor for the density function is given by *e*(*u*). The amount of smoothing (i.e., standard deviation) in Gaussian smoothing kernel *k* is defined by *σ* as follows: 

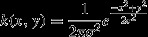
2

The core of a golden eagle territory does not typically extend more than 3 km from the nest, but eagles regularly visit sites further from their nest (McGrady et al. 2002), especially after the chicks have fledged. Therefore, the spatial extent within which the eagle predation might be strongest was tested by calculating the density maps at five different levels of *σ* smoothing, at 1–5 km. An optimal level of *σ* was then selected through model comparison.

### Game animal abundance data

The abundance data of game animals from the wildlife triangle scheme comprised of snow-track counts of several mammalian species from 1989 to 2011, derived from 1610 individual triangles (17,808 individual records) (Fig.1). Data were provided by the Finnish Game Research Institute, which coordinates the annual nationwide census performed by hunters. Triangle locations are fixed, and most, but not all, triangles are censused every year. The track counts are carried out mainly during February by monitoring the triangular 3 × 4 km transect lines, established throughout the country (Lindén et al. 1996; Pellikka et al. 2005). Old tracks are covered by snowfall or track counters, and after a sufficient track accumulation time, new tracks are counted. Typically, the results of the track counts are expressed as track density (crossings per 24 h per 10 km) (Pellikka et al. 2005), but in this study the original track count observations were used, complemented by a variable reporting the track accumulation time in days. Observations with an especially long accumulation time (>10 days) were discarded. Additionally, visual sightings of grouse are recorded during the census, and these abundance data were also employed in this study.

Abundance index data of two common mesopredator species, red fox and pine marten, were used as response variables. These two mesopredators are fairly active during winter and are thus well presented in the snow-track counts. The red fox, as a larger species, may also prey on the smaller pine marten (Lindström et al. 1995). The indices of mountain hare (*Lepus timidus*, L.), capercaillie (*Tetrao urogallus*, L.), black grouse (*Tetrao tetrix*, L.), hazel grouse (*Tetrastes bonasia*, L.), and willow grouse (*Lagopus lagopus*, L.) were also employed as explanatory prey covariates. Observations of all the bird species were summed together as the variable “grouse”. Track counts, when used as a covariate, were scaled with track accumulation time.

Using the midpoint coordinates of the wildlife triangles, the observed game abundances of an individual triangle were linked with the eagle densities extracted from the TD and FD raster maps at the same location. In a given year, the game observations were linked with TD of the same year and with FD from the previous year. The reason for this was that TD corresponds well to eagle density during the winter since adult eagles keep to their territories year-round, apart from the northernmost populations that are forced to migrate because of severe winter conditions (McGrady et al. 2002; Watson 2010). Meanwhile, FD is a good proxy for the hunting pressure posed by eagles during the summer and autumn, when the fledglings move about the territory and their parents hunt large amounts of food for their offspring and themselves (Watson 2010). Thus, FD would most likely affect the observed mesopredator abundances in the following winter.

In addition to the density variables, the distance to the closest active eagle nest from each wildlife triangle was calculated for each year. The variable “distance to nearest nest” (DNN) was then used as a competing explanatory eagle variable and compared with TD and FD. DNN increases linearly when moving further from the nest, and therefore, it may better describe the predation effect of eagle at the periphery of the territories. However, the effect of DNN is of interest only within distances within which eagles and mesopredators move about, as it is not plausible that eagles would reduce mesopredator numbers far outside their territory limits. Density variables TD and FD approach zero quite quickly when moving further away from the territory core area, but their advantage is that they are able to account for two or more territories close to each other, whereas DNN does not reflect the overall local eagle abundance well.

### Habitat data

As mesopredator abundances depend on landscape features, variables describing habitat composition were incorporated into our analyses. Habitat information was acquired from CORILIS data (raster size 1 × 1 km), which gives the proportion of different Corine Land Cover (CLC) classes within a smoothing radius of 5 km (European Environment Agency 2009). The data are derived from Landsat satellite imagery from 2000. The proportions of farmland and forest in the landscape were calculated by summing the proportions of CLC classes 12–22 and 23–29, respectively. Habitat proportions were then assigned to each wildlife triangle according to the raster cell in which the triangle midpoint was located, so that the variables give percentages of farmland and forest in the landscape within a 5 km radius from the triangle centers. The amount of farmland and forest habitat within landscape has previously been found to influence the abundances of red foxes and pine martens (Kurki et al. 1998).

Latitude and longitude variables were included in the models as they were expected to explain the impact of regionally varying environmental conditions upon the mesopredators. The productivity of the forests in northern Finland is distinctly lower than in the southern parts of the country (Peltola 2009). Related to this, conditions in northern Finland are harsher than in the south, as temperatures are lower and there is typically more snowfall during the dark polar winters. On the other hand, reindeer husbandry occurs only in the northern parts of Finland (Fig.1), and in this area, the diet of the golden eagle also includes reindeers, mainly in the form of calves and carcasses (Nybakk et al. 1999; Sulkava et al. 1999; Norberg et al. 2006).

### Statistical analyses

For data handling and analyses, we used R-software, version 3.0.2 (R Core Team 2013). Statistical analyses were conducted with generalized additive mixed model (GAMM), using the “gamm” function from the package “mgcv” (Wood 2011).

To analyze the impact of golden eagle upon the abundance indices of red fox and pine marten, we built models with quasi-Poisson distribution. DNN, and TD and FD with *σ* 1–5 km were all set as explanatory variables in separate models (11 models in total). The interaction of longitude and latitude was included in the models to account for large-scale spatial autocorrelation. Also, the following covariates were centered and scaled, following Schielzeth (2010), and included in the model structure: the proportion of farmland, the proportion of forest, mountain hare abundance, grouse abundance, and the time of mesopredator track accumulation in days. In the pine marten models, the abundance of the larger red fox was also included as a predator covariate. To account for repeated samples from the same triangles, that is, to handle pseudo-replication issues (Hurlbert 1984), a random structure of individual triangles and an AR1 correlation structure of triangle ID within year were included in all models.

Model selection was started by fitting a smoother to all covariates except the track accumulation day, which was expected to have a linear relation with the response variable. For other variables than eagle and coordinates, the *k* (upper limit on the degrees of freedom associated with a smooth) was constricted to improve convergence. From linear estimates, the unnecessary smoothers were removed and then the model was refitted. After this, all nonsignificant variables were removed – only eagle variables were always retained as a minimal model structure in order to improve the comparability of the models. The best *σ* for TD and FD was selected based on the amount of deviance explained in the final models (in TD models 4 km for red fox, 5 km for pine marten; in FD models 5 km for both species, see Table1). Results are reported only for models TD*σ*, FD*σ,* and DNN for red fox and pine marten (for variables retained in final models, see Table2).

**Table 1:** The amount of deviance explained (DE) in all red fox and pine marten models. Models selected based on their DE have bolded values

Red fox models	DE	Pine marten models	DE
Distance to nearest nest	**95145.24**	Distance to nearest nest	**19079.21**
Territory density, *σ* 4 km	**94824.11**	Territory density, *σ* 5 km	**18762.96**
Territory density, *σ* 3 km	94823.30	Territory density, *σ* 4 km	18750.45
Territory density, *σ* 5 km	94811.90	Territory density, *σ* 3 km	18668.46
Territory density, *σ* 2 km	94786.98	Territory density, *σ* 2 km	18639.04
Fledgling density, *σ* 5 km	**94778.21**	Fledgling density, *σ* 5 km	**18630.63**
Fledgling density, *σ* 4 km	94773.42	Fledgling density, *σ* 4 km	18607.4
Fledgling density, *σ* 3 km	94759.72	Fledgling density, *σ* 3 km	18597.67
Fledgling density, *σ* 1 km	94753.89	Fledgling density, *σ* 2 km	18581.58
Fledgling density, *σ* 2 km	94751.36	Fledgling density, *σ* 1 km	18568.67
Territory density, *σ* 1 km	94751.32	Territory density, *σ* 1 km	18567.63

**Table 2:** Retained variables in the selected GAMM models for red fox and pine marten. The variables applied with smoothers are in italics. Interactions are marked with a symbol ×. E and N refer to latitudinal and longitudinal coordinates and abbreviation acc.days to the snow-track accumulation time in days

Response	Predators	Location, habitat	Prey, census time
Red fox	Territory density	*E × N, farmland*,*forest*	*Mountain hare*, acc. days
Red fox	Fledgling density	*E × N, farmland*,*forest*	*Mountain hare*, acc. days
Red fox	*Distance to nearest nest*	*E × N, farmland, forest*	*Mountain hare*, acc. days
Pine marten	*Territory density, red fox*	*E × N, farmland,* forest	*Mountain hare*, acc. days
Pine marten	Fledgling density, *red fox*	*E × N, farmland,* forest	*Mountain hare*, acc. days
Pine marten	*Distance to nearest nest, red fox*	*E × N, farmland,* forest	*Mountain hare*, acc. days

## Results

### Red fox

**Figure 3:**
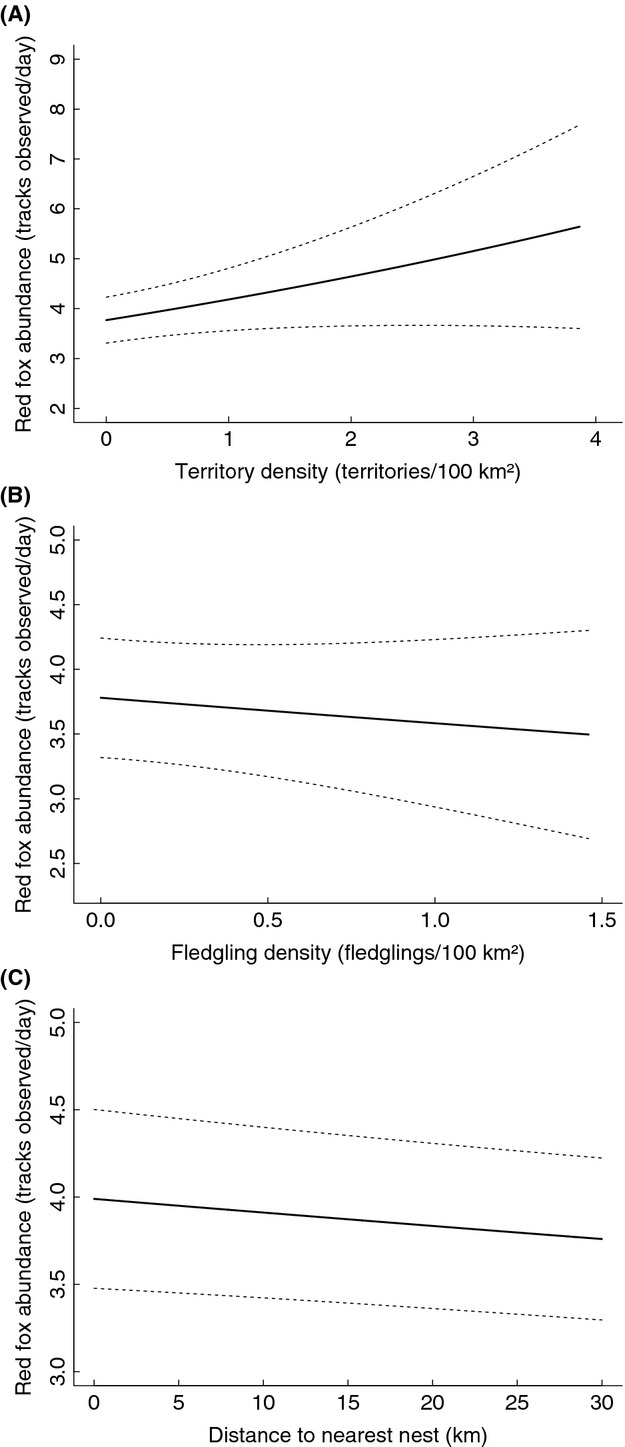
Red fox snow-track abundance estimates at varying golden eagle territory density (TD, panel A), fledgling density (FD, panel B), and distance to nearest nest (DNN, panel C), presented with 95% CI (dashed lines).

The relationship between the territory density (TD, *σ* 4 km) and red fox abundance was linear: the more eagles there were, the higher fox numbers were (*t* = 2.305, *P* = 0.021, Fig.3A). In contrast, fledgling density of eagles in the previous year (FD, *σ* 5 km) had no obvious association with fox abundance (*t* = −0.777, *P* = 0.437, Fig.3B). The influence of distance to nearest eagle nest (DNN) was nonlinear, but when examined only at close distances (up to 30 km) it was fairly linear: the shorter the distance to active eagle nests was, the more foxes there were (*F* = 6.337, *P* < 0.001, Fig.3C).

The impact of habitat variables on red foxes was the same in all three models (see Table S1 for all results). Intermediate proportions of farmland and low to intermediate proportions of forest in the landscape were most beneficial for foxes. As expected, the abundance of foxes increased with increasing mountain hare numbers, although this association leveled off at very high hare abundances. In spatial terms, the model predicted highest fox abundances in agriculture- and forest-dominated southwest Finland, gradually decreasing toward northeast, with a local decrease in the eastern parts of Middle Finland.

### Pine marten

**Figure 4:**
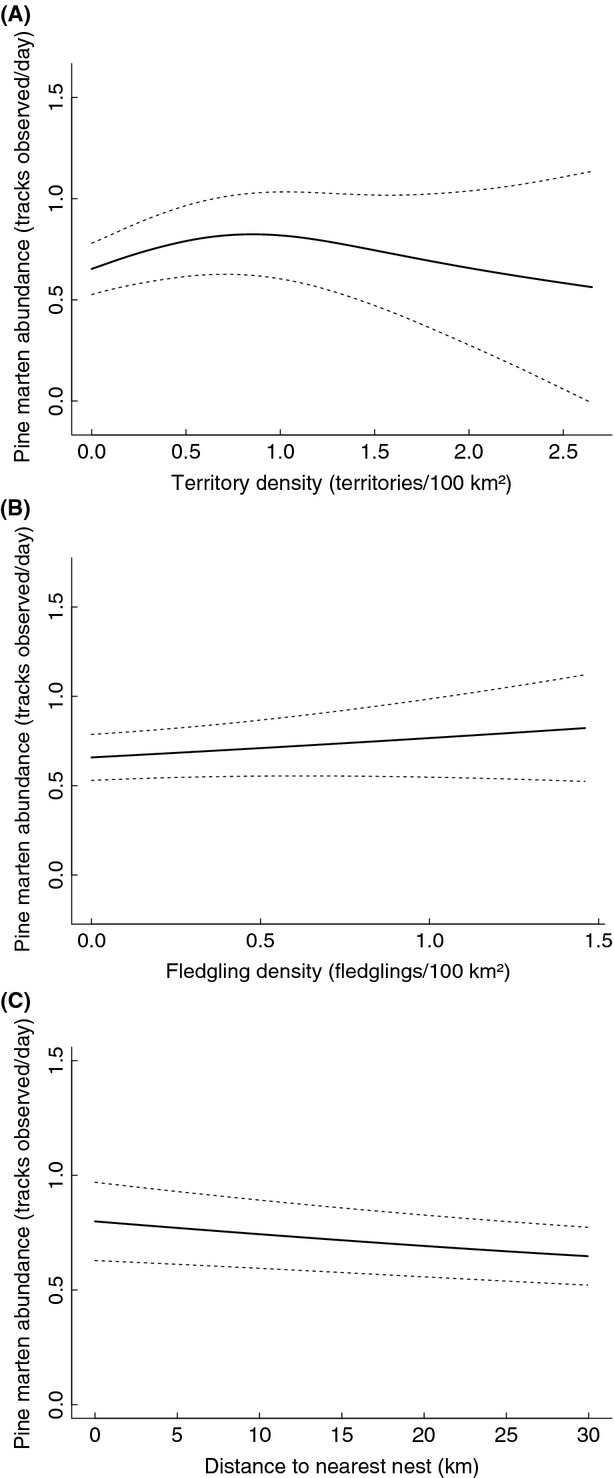
Pine marten snow-track abundance estimates at varying golden eagle territory density (TD, panel A), fledgling density (FD, panel B), and distance to nearest nest (DNN, panel C), presented with 95% CI (dashed lines).

Territory density (TD, *σ* 5 km) had a nonlinear relationship with pine marten abundance: from low to intermediate TD, the amount of martens slightly increased, but at high TD, it appeared to decrease (*F* = 5.300, *P* = 0.004, Fig.4A). However, wider confidence intervals resulting from relatively few data points at high eagle densities necessitate caution when interpreting the association. Fledgling density (FD, *σ* 5 km) had no obvious association with the marten abundances (*t* = 1.412, *P* = 0.158, Fig.4B). Distance to nearest nest (DNN) had a nonlinear impact on pine marten abundance, which at close range to active eagle nests translated to the shorter the distance, the more martens there were (*F* = 5.008, *P* < 0.001, Fig.4C).

**Figure 5:**
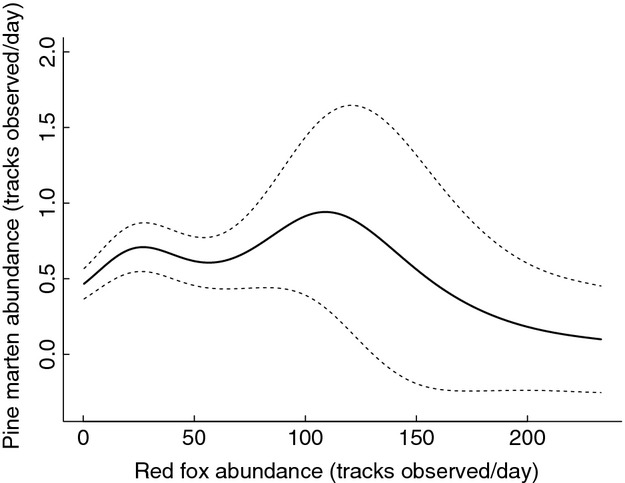
Pine marten snow-track abundance estimates at varying red fox abundances, retrieved from the distance to nearest nest (DNN) model. 95% CI are presented with dashed lines.

The response to red fox abundance was significant and identical in all of the three pine marten models: the number of martens observed increased slightly when the fox abundance increased from low to intermediate fox abundance, but at high fox abundances the number of martens decreased (*F* = 23.695, *P* < 0.001, Fig.5). To be conservative, the results are reported from the DNN-model, which had the lowest *F*-value. Moreover, all three models predicted pine marten abundances to be highest when the proportion of forest in the landscape was high (see Table S1 for all results). The response to proportion of farmland was nonlinear, with lowest marten abundances occurring at landscapes with intermediate amounts of farmland. Spatially examined the highest marten abundances occurred in southern and central Finland and the lowest in the western parts of Middle Finland.

## Discussion

We examined the abundances of two mammalian mesopredators, the red fox, and the pine marten, in relation to the predation risk imposed by the golden eagle by combining long-term data from monitoring of game animal abundances and eagle nesting. We predicted that marten abundance indices would be lower in areas where there are more active eagle territories and fledglings produced, and also when close to inhabited eagle nests, whereas red fox abundances would not respond negatively to these factors. Our results show that both mesopredators were most abundant in areas of high territory density and very close to active eagle nests. In regards to the pine marten, this was the opposite of our prediction. However, the results gave some indication that eagles could be harmful for pine martens at very high territory densities.

According to our results, red foxes seem to be abundant in the vicinity of active golden eagle nests. Although the amount of forest and farmland in the landscape was accounted for, we suspect that there were still some unexplained factors that both eagles and foxes benefit from, resulting in increased densities of both species in the same areas. The golden eagle is sensitive to any human disturbance (Ollila and Koskimies 2006; Kaisanlahti-Jokimäki et al. 2008), and the red fox can manage well in remote areas too, although it benefits from fragmented landscapes occurring close to human inhabitation (Kurki et al. 1998). Foxes and eagles prey partly on same prey species, such as hares, grouse, and other birds (Kauhala et al. 1998; Sulkava et al. 1999; Dell'Arte et al. 2007). The amount of hare and grouse was taken into account, but sufficient availability of other shared prey could enable the two species to thrive in same areas. For example, the local availability of reindeer carcasses would profit both species. All in all, it seems that at the population level foxes are able to avoid the harmful impacts of eagle predation or intimidation, as they did not respond to the landscape of fear modeled by eagle presence. This can result from the fact that red foxes are usually of the same weight or even larger than golden eagles, whereas in IGP, the killer is typically clearly larger than the prey (Palomares and Caro 1999; Sergio and Hiraldo 2008).

Similarly to the red fox, the abundance of pine marten was found to correlate positively with eagle territory density, specifically when moving from low to intermediate densities. This likely derives from the fact that the golden eagle and the pine marten benefit from similar habitats. Although the amount of forest coverage was controlled for in the analyses, there is also variation between forest stands in terms of wood volume, tree density, and stand age. Martens reside mainly in forested areas and prefer to move in old spruce- and pine-dominated stands (Brainerd and Rolstad 2002). Golden eagles typically nest in large pines (Ollila and Koskimies 2006), but habitats providing these trees are limited in Finnish forests. Thus, excluding very dense forest stands that are difficult for eagles to move in, the two species likely reside in similar type of habitats. This was also supported by the fact that we observed higher marten abundances within shorter distances from active eagle nests.

A density-dependent predation impact was found with red foxes on pine martens: at high abundances, foxes could act as an IG predator for marten. Red foxes have been observed to prey on pine martens, for example, in Sweden, where the decrease of foxes in 1980s due to sarcoptic mange, caused by a parasitic mite, was followed by an increase in the number of martens (Lindström et al. 1995). However, landscape-level studies from Fennoscandia have reported contrary results (Kurki et al. 1998). Considering red foxes and pine martens, it is important to note that these generalist predators may also compete over the same food resources, which likely intensifies their interaction at high abundances. Diet studies have shown that the overall prey selection for the two species is overlapping, although martens have less large-sized prey in their diet than foxes do (Pulliainen and Ollinmäki 1996; Kauhala et al. 1998).

Our results emphasize the importance of the timing of observations; in contrast to territory densities from the same time period, mesopredator abundances did not respond to the fledgling density of eagles in the previous summer. One reason for this could be that after fledging, juvenile eagles move outside the home range to such an extent that a clear predation impact within the territory cannot be observed. In addition, young eagles typically start dispersing already during late autumn (Watson 2010), after which the predation impact is generated solely by the adults remaining at the territory. Furthermore, movements of the mesopredator prey can dilute the effect of eagle predation. Therefore, a time lag of over 6 months may be too long for any predation impact to show on mesopredator abundances. Instead, it could be better to study the impact of breeding eagles and their fledglings on mesopredator abundances in autumn. Estimated eagle territory density, which is able to account for multiple close territories, was perhaps the best proxy for predation risk, although the distance to eagle nest explained the largest amount of variation in the data.

We expected to detect a predation impact of golden eagle upon pine marten abundance, but the analyses did not provide substantial support for this. However, we wish to note that the data showed a decreasing trend in marten abundance at high territory densities of eagles, that is, when there is one or more resident eagle pair within the area. As the impact of foxes was separately controlled for in our analyses, we do not believe that this trend was a result of combined predation pressure by eagle and fox. Instead, a response to the landscape of fear shaped solely by the eagle remains a more plausible explanation. After all, martens weigh approximately only one-third of the weight of the eagle and are thus an easier prey for the eagle to hunt than foxes are. It has previously been noted that pine martens avoid very open areas and clear cuts (Brainerd and Rolstad 2002), and this local-scale habitat preference of martens could be related to the threat of golden eagle predation (Korpimäki and Nordström 2004). In general, medium-size predators actively avoid habitats utilized by apex predators (Fedriani et al. 1999; Mukherjee et al. 2009). Similarly, the eagle could, in addition to direct killing, reduce marten numbers by intimidation. Also, eagles may disturb martens and hamper their foraging, hence decreasing their fitness. In an earlier study, predation risk imposed by the white-tailed sea eagle (*Haliaeetus albicilla*, L.) has been shown to reduce the swimming trips of another mustelid species, the American mink (*Neovison vison*, Schreber) (Salo et al. 2008). In contrast, strong food competition among golden eagles and pine martens is unlikely, as the diet of these species is fairly broad yet not overlapping much (Pulliainen and Ollinmäki 1996; Watson 2010). Research conducted at finer spatial scales could help to establish whether an abundant eagle population causes any reduction in pine marten numbers, for example, via behavioral changes that result in lower fitness. New information on the impact of the golden eagle could be acquired, for example, by radio-tracking habitat use of martens and observing the predation behavior of eagles.

When abundant, mesopredators hold the potential to induce both ecological and economic costs with their outbreaks (Prugh et al. 2009), and therefore, understanding processes related to their abundance is important. Species such as the red fox, pine marten, and American mink are suggested to limit or be the cause of decline of hare and bird populations (Marcström et al. 1988; Lindström et al. 1994; Kauhala and Helle 2000; Nordström et al. 2002). Not surprisingly, IGP from a top predator upon mesopredators may also cause cascading impacts on herbivores (e.g., Henke and Bryant 1999; Helldin et al. 2006; Prugh et al. 2009). Elmhagen et al. (2010) showed that when the recolonizing lynxes (*Lynx lynx*, L.) limit red fox populations, this has an indirect positive impact on mountain hare abundance. Fielding et al. (2003) suggested that through suppressing medium-sized raptors the golden eagle could reduce the overall predation pressure on game species. However, such cascading effects with avian–mammalian IGP have not really been examined, even though they could have extensive influence on ecosystems. For example, on the California Channel Islands (USA), the introduction of an exotic prey has enabled the golden eagle to strongly reshape the local mesopredator community and indirectly affect the granivore prey (Roemer et al. 2009). In northern Europe, the eagle owl (*Bubo bubo*) could also be acting as prominent avian top predator (Korpimäki and Nordström 2004).

Based on the results presented here, it is not clear whether herbivores (e.g., hares and grouse) would benefit from eagles preying upon and intimidating red fox and pine marten. Nonetheless, it is important to keep in mind that the Finnish mesopredator guild entails several other species that may be influenced by eagles. Moreover, by expanding their range in the future, golden eagles (Ollila and Ilmonen 2009; Watson 2010) as well as other raptors may increase predation on mesopredators in new areas. This could, in turn, improve the protection status of golden eagles and raptors in general if raptor-induced mesopredator suppression were to benefit herbivorous species. In addition, the predation impacts of raptors could be compared with those caused by mammalian top predators, which in Finland include the increasingly numerous lynx and bear (*Ursus arctos*), as well as the less common and probably only locally relevant wolf and wolverine (*Gulo gulo*, L.) (Wikman 2010). This type of comparison of the strength of top-down suppression would help to assess the importance of raptors in boreal ecosystems.

## Conclusions

This study provides novel information on avian–mammalian IGP by examining the relationship between the golden eagle and its potential prey, the pine marten and the red fox. We found both mesopredator species to be most abundant in proximity to the golden eagle suggesting that killing and intimidation by eagles may not be a relevant cause of decrease for populations of martens and foxes. However, we surmise that pine martens could suffer from predation at high densities of eagle territories, particularly due to their smaller size. The information reported in this article improves our understanding of the role of avian top predators in terrestrial communities, but further long-term studies are required to form a clearer picture of the impacts that recovering avian top predators, such as the golden eagle, have on predator guilds. Gathering behavioral data would also help us to understand in more detail how mesopredators cope within the presence of large avian predators. Potential cascading effects of mesopredator suppression on lower trophic levels would be of great interest, too.

## Data Accessibility

Data used for this study can be found in Dryad data archive (ASCII text file, doi: 10.5061/dryad.qc6nv). The location data are not included to avoid endangering nesting sites of the golden eagle. Habitat data come from the European Environment Agency (2009).

## Supporting Information

**Table S1.** The results of selected GAMM models for red fox and pine marten.
