# Supplementary material for: Avian top predator and the landscape of fear: responses of mammalian mesopredators to risk imposed by the golden eagle
Source: Ecol Evol. 2015 Jan 5;5(2):503–14. doi: 10.1002/ece3.1370 (PMC4314280; doi:10.1002/ece3.1370)
Supplement: Supplementary file 1 [file ece30005-0503-sd1.pdf]

SUPPLEMENTARY INFORMATION

Avian top predator and the landscape of fear: responses of mammalian mesopredators to risk imposed by the golden eagle

Mari S. Lyly*, Alexandre Villers, Elina Koivisto, Pekka Helle, Tuomo Ollila & Erkki Korpimäki
*Corresponding author: Tel: +358 2333 5813; Fax +358 2333 6568; E-mail: mari.s.lyly@utu.fi

Table S1. The results of selected GAMM models for red fox and pine marten. Linear and additive variables are reported separately. Abbreviation acc.days refers to snow-track accumulation time in days, letters E and N denote the latitudinal and longitudinal coordinates, and DNN is the distance to nearest eagle nest. Letter σ is the amount of smoothing in the Gaussian kernel when calculating the territory and fledgling densities of golden eagle. EDF stands for estimated degrees of freedom.

Red fox

**Territory density (σ 4km)**	**Fixed effects**	**Estimate**	**t**	**P**
	intercept	1.858	146.014	<0.001
	territory density	0.022	2.305	0.0212
	acc.days	0.126	22.961	<0.001
	**Additive effects**	**EDF**	**F**	**P**
	s(E,N)	25.27	40.019	<0.001
	s(farmland)	2.44	8.413	<0.001
	s(forest)	1.62	15.326	<0.001
	s(mountain hare)	4.93	408.155	<0.001
**Fledgling density (σ 5km)**	**Fixed effects**	**Estimate**	**t**	**P**
	intercept	1.858	146.064	<0.001
	fledgling density	-0.006	-0.777	0.437
	acc.days	0.126	22.979	<0.001
	**Additive effects**	**EDF**	**F**	**P**
	s(E,N)	25.30	40.206	<0.001
	s(farmland)	2.42	8.112	<0.001
	s(forest)	1.58	15.483	<0.001
	s(mountain hare)	4.93	408.331	<0.001
**Distance to nearest nest**	**Fixed effects**	**Estimate**	**t**	**P**
	intercept	1.858	146.44	<0.001
	acc.days	0.126	22.93	<0.001
	**Additive effects**	**EDF**	**F**	**P**
	s(DNN)	3.33	6.337	<0.001
	s(E,N)	25.35	37.71	<0.001
	s(farmland)	2.76	8.055	<0.001
	s(forest)	1.47	15.756	<0.001
	s(mountain hare)	4.93	409.588	<0.001

Pine marten

**Territory density (σ 5km)**	**Fixed effects**	**Estimate**	**t**	**P**
	intercept	0.102	5.121	<0.001
	forest	0.080	3.219	0.001
	acc.days	0.197	21.868	<0.001
	**Additive effects**	**EDF**	**F**	**P**
	s(territory density)	2.16	5.3	0.004
	s(E,N)	24.43	14.506	<0.001
	s(red fox)	3.94	23.763	<0.001
	s(farmland)	3.27	9.155	<0.001
	s(mountain hare)	4.70	86.185	<0.001
**Fledgling density (σ 5km)**	**Fixed effects**	**Estimate**	**t**	**P**
	intercept	0.102	5.102	<0.001
	fledgling density	0.017	1.412	0.158
	forest	0.079	3.159	0.002
	acc.days	0.197	21.88	<0.001
	**Additive effects**	**EDF**	**F**	**P**
	s(E,N)	24.58	14.2	<0.001
	s(red fox)	3.94	24.75	<0.001
	s(farmland)	3.31	9.39	<0.001
	s(mountain hare)	4.70	86.03	<0.001
**Distance to nearest nest**	**Fixed effects**	**Estimate**	**t**	**P**
	intercept	0.101	5.056	<0.001
	forest	0.084	3.38	<0.001
	acc.days	0.196	21.817	<0.001
	**Additive effects**	**EDF**	**F**	**P**
	s(DNN)	4.78	5.008	<0.001
	s(E,N)	24.24	11.309	<0.001
	s(red fox)	3.95	23.695	<0.001
	s(farmland)	3.24	8.593	<0.001
	s(mountain hare)	4.70	86.491	<0.001
